# Twinning behavior of orthorhombic-α” martensite in a Ti-7.5Mo alloy

**DOI:** 10.1080/14686996.2019.1600201

**Published:** 2019-04-30

**Authors:** Xin Ji, Ivan Gutierrez-Urrutia, Satoshi Emura, Tianwei Liu, Toru Hara, Xiaohua Min, Dehai Ping, Koichi Tsuchiya

**Affiliations:** a Research Center for Structural Materials, National Institute for Materials Science, Tsukuba, Ibaraki, Japan; b School of Materials Science and Engineering, Dalian University of Technology, Dalian, P.R. China; c Graduate School of Pure and Applied Sciences, University of Tsukuba, Tsukuba, Ibaraki, Japan

**Keywords:** β-Titanium alloys, orthorhombic-α” martensite, deformation twinning, transformation twinning, transmission electron microscopy, 10 Engineering and Structural materials, 106 Metallic materials, 303 Mechanical / Physical processing, 503 TEM, STEM, SE, 504 X-ray / Neutron diffraction and scattering

## Abstract

Deformation microstructure of orthorhombic-α” martensite in a Ti-7.5Mo (wt.%) alloy was investigated by tracking a local area of microstructure using scanning electron microscopy, electron back-scattered diffraction, and transmission electron microscopy. The as-quenched α” plates contain {111}_α”_-type I transformation twins generated to accommodate transformation strain from bcc-β to orthorhombic-α” martensite. Tensile deformation up to strain level of 5% induces {112}_α”_-type I deformation twins. The activation of {112}_α”_-type I deformation twinning mode is reported for the first time in α” martensite in β-Ti alloys. {112}_α”_-type I twinning mode was analyzed by the crystallographic twinning theory by Bilby and Crocker and the most possible mechanism of atomic displacements (shears and shuffles) controlling the newly reported {112}_α”_-type I twinning were proposed.

## Graphical abstract

**Figure:**
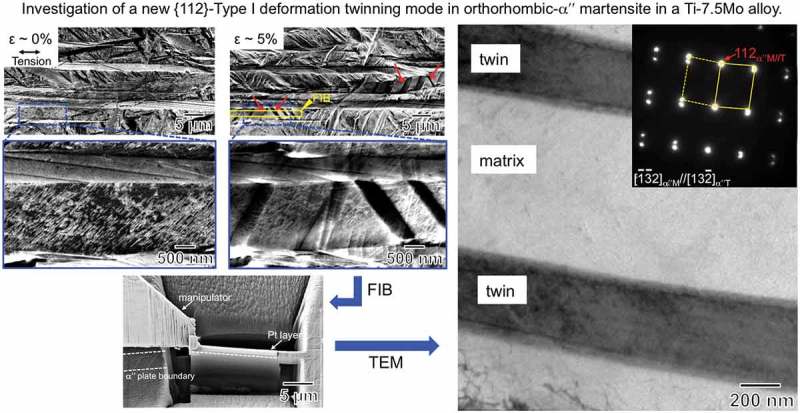

## Introduction

β titanium alloys such as Ti-Mo, Ti-Nb, and Ti-Ta alloys have attracted considerable attention for biomedical applications owing to their excellent properties such as low Young’s modulus, superior biocompatibility, high corrosion resistance, shape memory effect, and superelasticity [1–3]. One characteristic of these materials is the martensitic transformation from body centered cubic (bcc)-β to orthorhombic-αʺ martensite upon quenching or deformation, which strongly depends on the content of β-stabilizing elements such as Mo, Nb, and Ta [4–7]. αʺ martensite has been reported to play a key role in several mechanical properties of β-Ti alloys [7–9]. For example, the shape memory effect and the superelasticity of Ti-Nb alloys are associated with their reversible martensitic transformation between β and αʺ martensite [7,10]. Meanwhile, alloys with αʺ martensite structure exhibit lower Young’s modulus close to the values of human bones (~30 GPa), which is essential for the design of advanced biomedical materials [4,11–13]. In particular, Ti-7.5Mo (wt.%) alloy with full αʺ martensite structure exhibits a low Young’s modulus of ~65 GPa [4] and good bone-implant interaction [14]. Therefore, the Ti-7.5Mo alloy has been regarded as a promising candidate for implant applications [15,16]. Extensive studies have been conducted on the crystal structure of αʺ martensite in β-Ti alloys [17–20]. These studies reveal that αʺ martensite contains internal twin structure, namely, transformation twin structure, which is associated with transformation strain accommodation from β to αʺ martensite [19,21–24]. Different transformation twinning modes have been reported such as {111}_αʺ_-type I [19,22–24], <211>_αʺ_-type II [19,25], and {011}_α”_-compound twinning modes [19,26,27]. The activation of these twinning modes strongly depends on the lattice parameters of β and αʺ martensite [19,28]. For instance, Inamura et al. [19] reported the activation of different twinning modes in Ti-(33–46)Nb-3Al (wt.%) alloys, namely {111}_α”_-type I, <211>_αʺ_-type II and {011}_αʺ_-compound twinning, which is associated with Nb content.

Despite the advantages of low Young’s modulus and good biocompatibility, Ti-Mo alloys with αʺ martensite structure exhibit a major drawback, which is the relatively low mechanical strength compared to β-Ti alloys with single β phase [29,30]. Accordingly, a better understanding of deformation mechanisms of αʺ martensite is essential for the development of high-performance alloys [6,31–33]. So far, few studies have analyzed deformation behaviors of αʺ martensite in β-Ti alloys [25,34,35]. These studies have reported {130}_αʺ_-compound twinning in a Ti-25Ta-20Nb (wt.%) alloy [25], and {130}_αʺ_-compound and {103}_αʺ_-compound twinning in a Ti-42Nb (wt.%) alloy [34]. Therefore, the aim of this study is to investigate the deformation behaviors of αʺ martensite in Ti-Mo alloys. A Ti-7.5Mo (wt.%) alloy with αʺ martensite structure was prepared. We have performed tensile tests and tracked deformation microstructure of as-quenched αʺ martensite by combining scanning electron microscopy (SEM) and transmission electron microscopy (TEM). Deformation twin structure was analyzed by the crystallographic twinning theory of Bilby and Crocker [36,37]. The possible mechanisms of atomic movements, that is, shears and shuffles, controlling the active twinning modes were discussed.

## 2. Experimental details

The Ti-7.5Mo alloy was prepared by cold crucible levitation melting under Ar gas atmosphere. The ingot was hot forged at 1273 K to 40% thickness reduction and thereafter hot rolled at 1173 K to 75% thickness reduction followed by air-cooling. The hot-rolled material was subsequently solution-treated for 1 h at 1173 K followed by water quenching. Tensile tests were conducted in an INSTRON 5581 (Instron, Japan) testing machine at room temperature with an initial strain rate of 2.8 × 10^−4^ s^−1^. Flat dog-bone-shaped tensile specimens with gauge section of 18 mm (length) × 4 mm (width) × 2 mm (thickness) were cut by electro-discharge machining. Phase analysis was carried out by X-ray diffraction (XRD) in a RINT-TTR III (Rigaku, Japan) diffractometer with Cu-K_α_ radiation. Microstructure characterization was performed by backscattered electron imaging (BSE) and electron backscattered diffraction (EBSD) in a Zeiss Σigma field emission gun SEM (Carl Zeiss AG, Germany) equipped with a TSL (TSL, EDAX, USA) orientation imaging microscopy (OIM) system. EBSD maps were obtained at 20 kV with step sizes ranging between 20 nm and 0.5 μm. TEM observation was carried out by a JEM-2100F (JEOL, Japan) microscope operated at 200 kV. TEM thin foils were prepared by twin-jet electropolishing with a solution of methanol, 1-butanol, and perchloric acid (13:6:1 in volume) at 238 K and at a voltage of 20 V.

**Figure 1.**
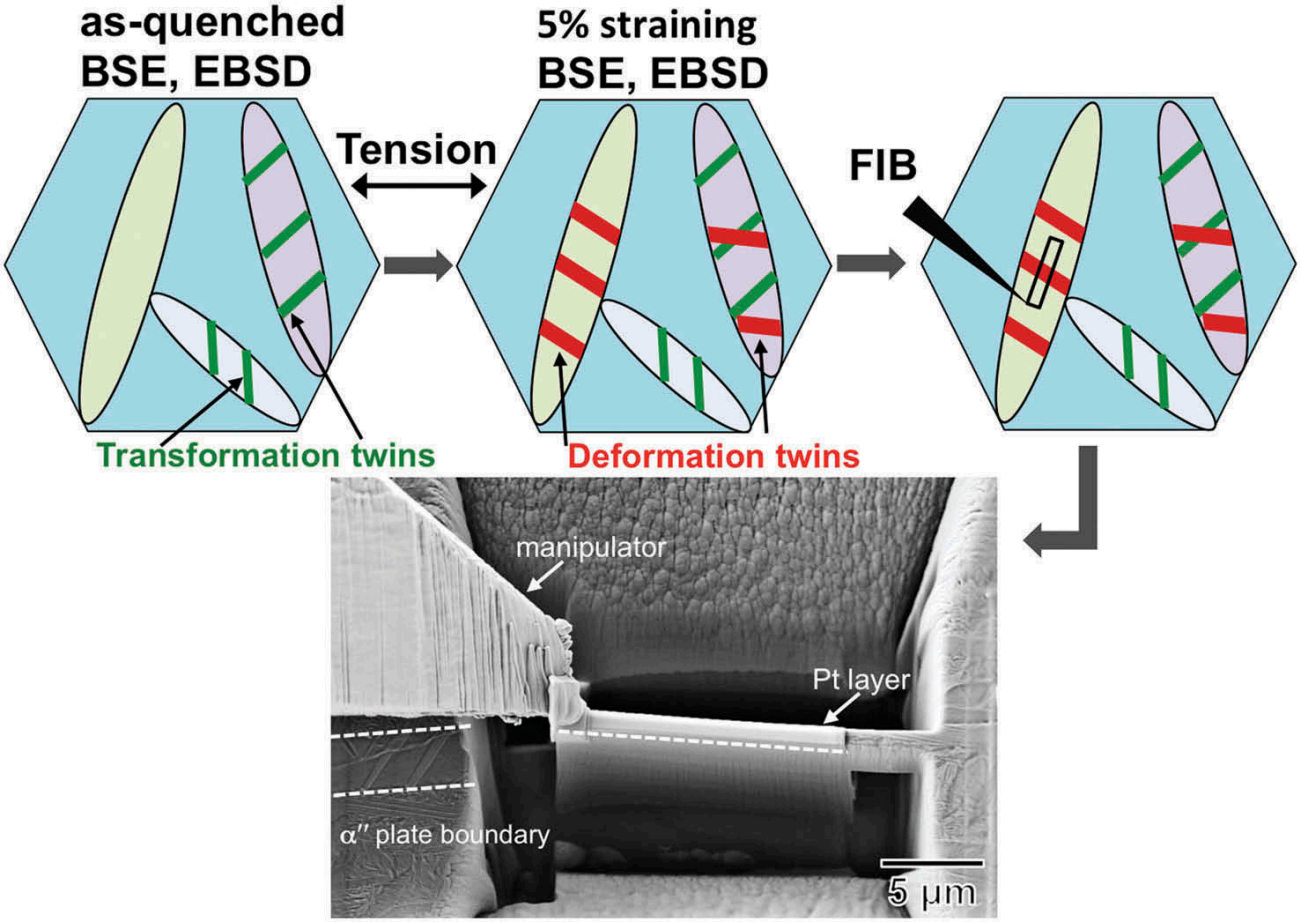
Experimental approach for the characterization of deformation microstructure, and secondary electron (SE) image of in-depth TEM lamella cut from the deformation twins.

Deformation mechanisms of as-quenched αʺ martensite were investigated by a combined SEM-TEM approach, Figure 1. A microstructural area of as-quenched state was firstly evaluated by BSE and EBSD. Subsequently, the sample was deformed upon 5% straining with the tensile axis (TA) parallel to the horizontal line. This area was tracked by BSE and EBSD without any additional grinding or polishing. Thereafter, a TEM sample was cut from the tracked αʺ martensite plates and lifted out by a manipulator by using a dual-beam focused ion beam (FIB) in a FIB-SEM Zeiss Auriga instrument. A Pt layer was deposited on the sample surface before FIB milling to avoid any surface damage.

## 3. Results

### 3.1. Phase analysis

**Figure 2.**
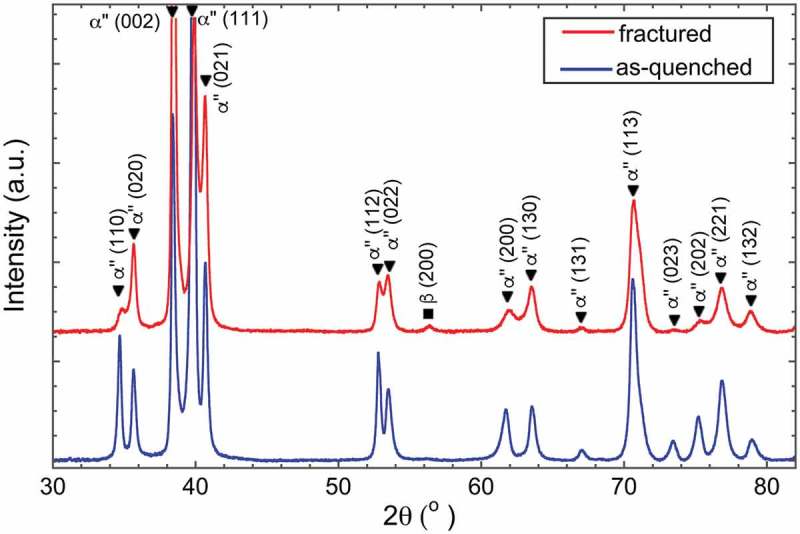
X-ray diffraction profiles of Ti-7.5Mo alloy in as-quenched (blue line) and tensile fractured (red line) states.

Figure 2 shows XRD profiles of the Ti-7.5Mo alloy in as-quenched and fractured (tensile strain ε ~ 19% [38]) states. It can be seen that the as-quenched alloy mainly consists of orthorhombic-αʺ martensite phase although prior bcc-β phase is still present, as indexed from the (200)_β_ reflection at 2*θ* = 55.8°. The lattice parameters of αʺ martensite were determined as *a*_αʺ_ = 0.3002 ± 0.0001 nm, *b*_αʺ_ = 0.5033 ± 0.0005 nm, and *c*_αʺ_ = 0.4680 ± 0.0005 nm. After tensile fracture, the positions of diffraction peaks remain unchanged. This result suggests that in the present alloy, phase transformation did not take place upon tensile deformation.

### 3.2. Microstructure characterization

#### 3.2.1. As-quenched state

**Figure 3.**
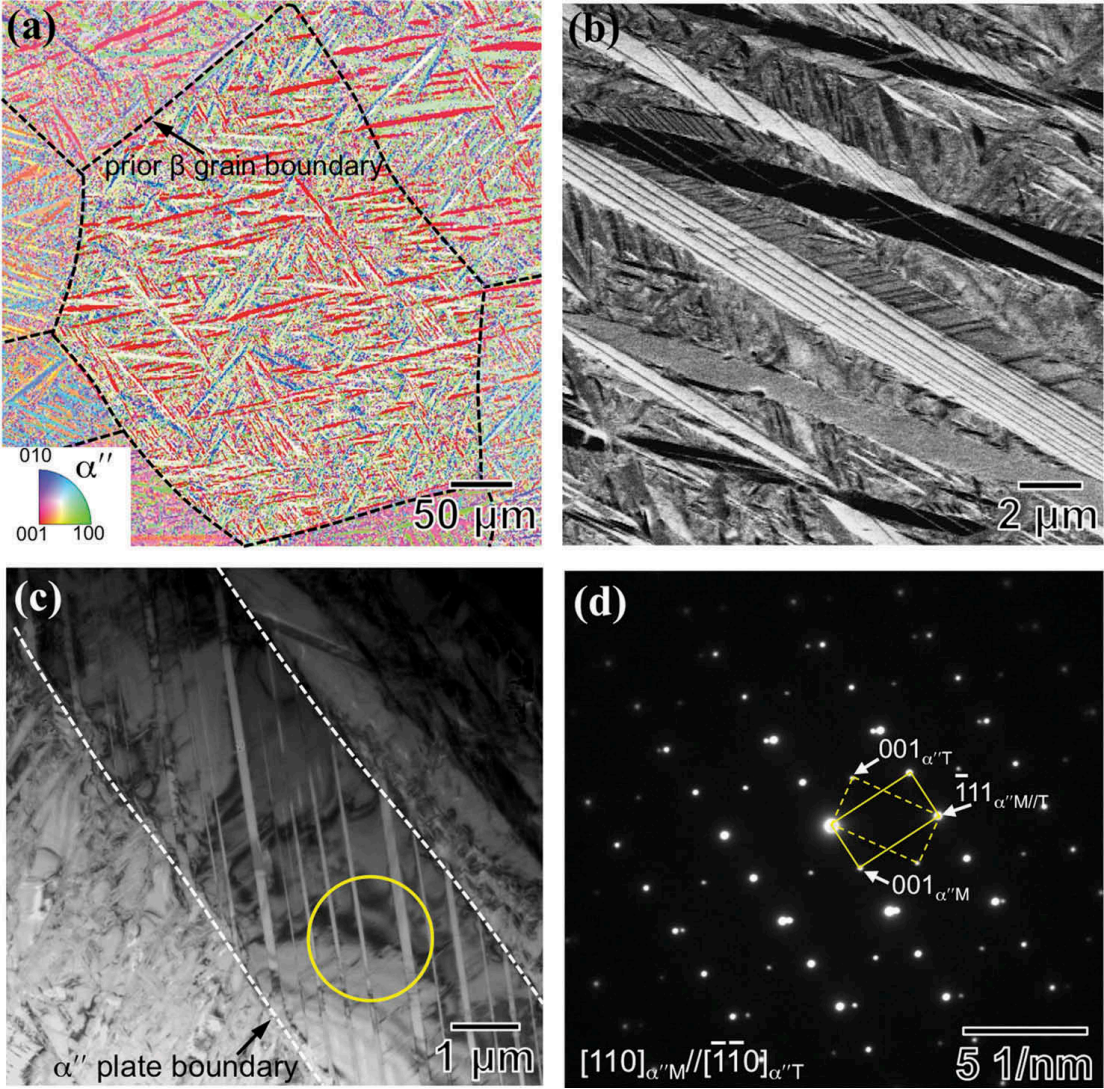
(a) Inverse pole figure (IPF) map of the as-quenched microstructure with dashed lines indicating the prior β grain boundaries. (b) Backscattered electron (BSE) image showing the acicular αʺ plates. (c) TEM image of αʺ martensite with internal twinning structure. (d) Selected area electron diffraction (SAED) pattern of the circled area in (a). The reflections connected by the yellow solid and dashed lines originate from matrix and twin, respectively. The subscripts ‘M’ and ‘T’ denote matrix and twin, respectively.

EBSD-inverse pole figure (IPF) map of Figure 3(a) shows the acicular morphology of αʺ martensite plates, which are uniformly distributed throughout the prior β grain. This figure also reveals a small content of β phase (area fraction <5%), which agrees with XRD data. It can be seen that αʺ martensite contains average thickness of ~ 2 μm and most of martensite plates exhibit a well-defined internal twin structure (Figure 3(b)). A detailed TEM analysis was performed on an αʺ martensite plate containing internal twins, Figure 3(c). Selected area electron diffraction (SAED) pattern exhibits two sets of reflections from [110]_α"M_ and [$\overset{ˉ}{1}\overset{ˉ}{1}0$]_α"T_ crystallographic orientations (M: matrix; T: twin), which corresponds to a mirror symmetry with respect to the $\left(\overset{ˉ}{1}11\right)$_α"M//T_ plane, Figure 3(d). This result indicates that the internal twin structure of αʺ martensite plates contains a {111}_αʺ_ twin plane. Accordingly, these twin plates correspond to {111}_αʺ_-type I twins [39]. Since these twins are reported to accommodate the transformation strain during β to αʺ martensitic transformation [19,24], they are hereafter referred to as {111}_αʺ_-type I transformation twinning.

#### 3.2.2. Deformation state

**Figure 4.**
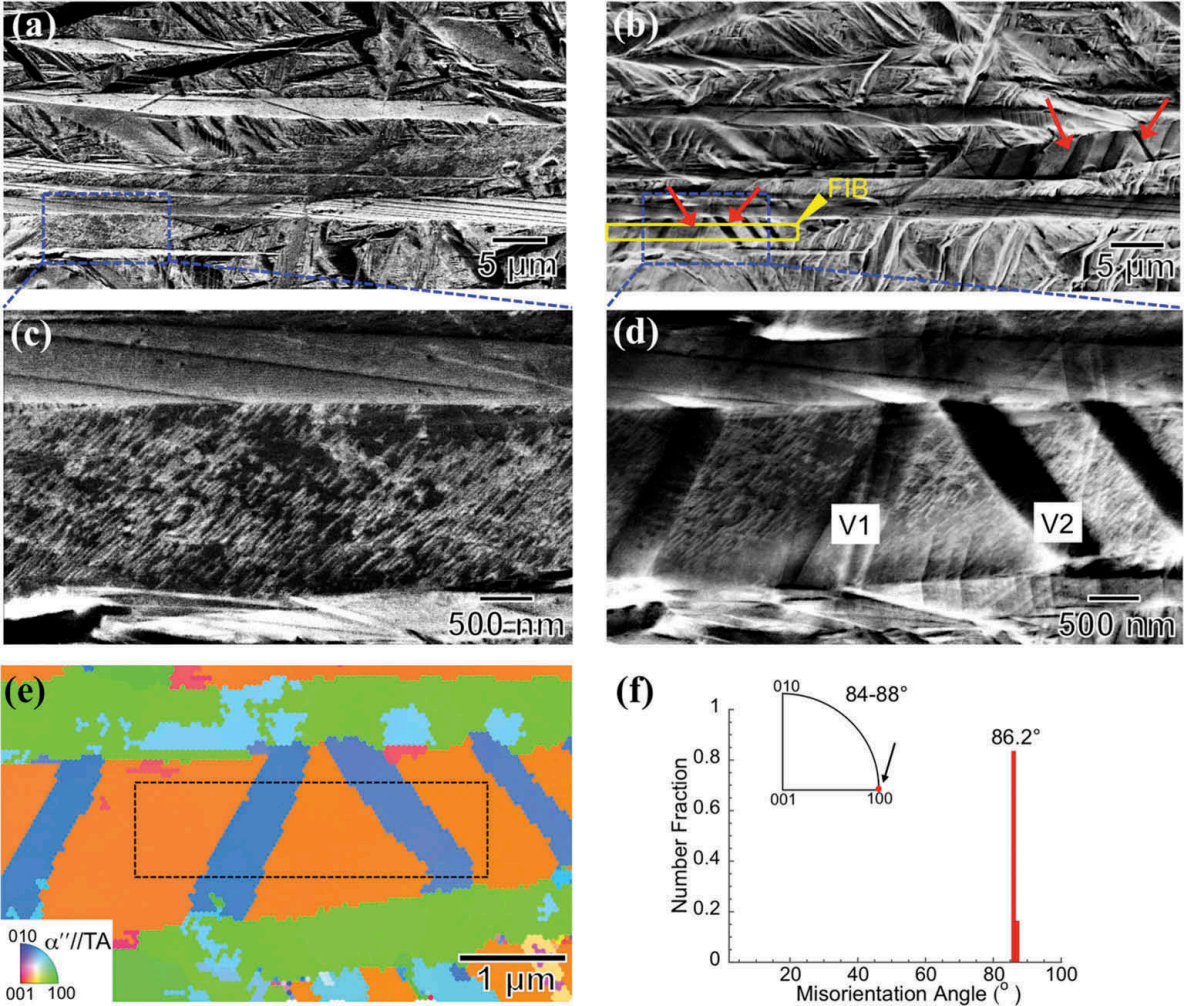
Sequential BSE images showing the microstructures of the as-quenched state, (a), and after 5% straining, (b). The tensile axis (TA) direction is horizontal respect to the images. The deformation bands are indicated by red arrows. Higher magnification images of (c) and (d) are taken from the boxed regions in (a) and (b), respectively. (e) Corresponding IPF image of (d). (f) Distributions of misorientation angle and misorientation axis (in the crystal coordinate) from the cropped region shown in (e).

We have tracked a typical area of αʺ martensite upon tensile deformation, Figure 4. Figure 4(a) shows the microstructure of the tracked area in the as-quenched state. After 5% tensile straining, several deformation bands are formed throughout martensite plates, as indicated by the red arrows in Figure 4(b). Figure. 4(c,d) correspond to a high magnified area containing three αʺ plates, from the framed regions in Figure. 4(a,b), respectively. Two variants of deformation bands (V1 and V2) are seen to nucleate at one αʺ plate boundary and then propagate to the other side of the boundary, Figure 4(d). EBSD analysis shows that V1 and V2 have the same crystallographic structure as αʺ matrix, Figure 4(e). The misorientation angle distribution and misorientation axis distribution plots are shown in Figure 4(f). It can be observed that both deformation bands have a misorientation angle of 86.2 ± 0.5° with respect to the αʺ matrix. The misorientation axis is shown around the <100>_αʺ_ direction (indicated by the black arrow) for the misorientation angle of 84–88°.

**Figure 5.**
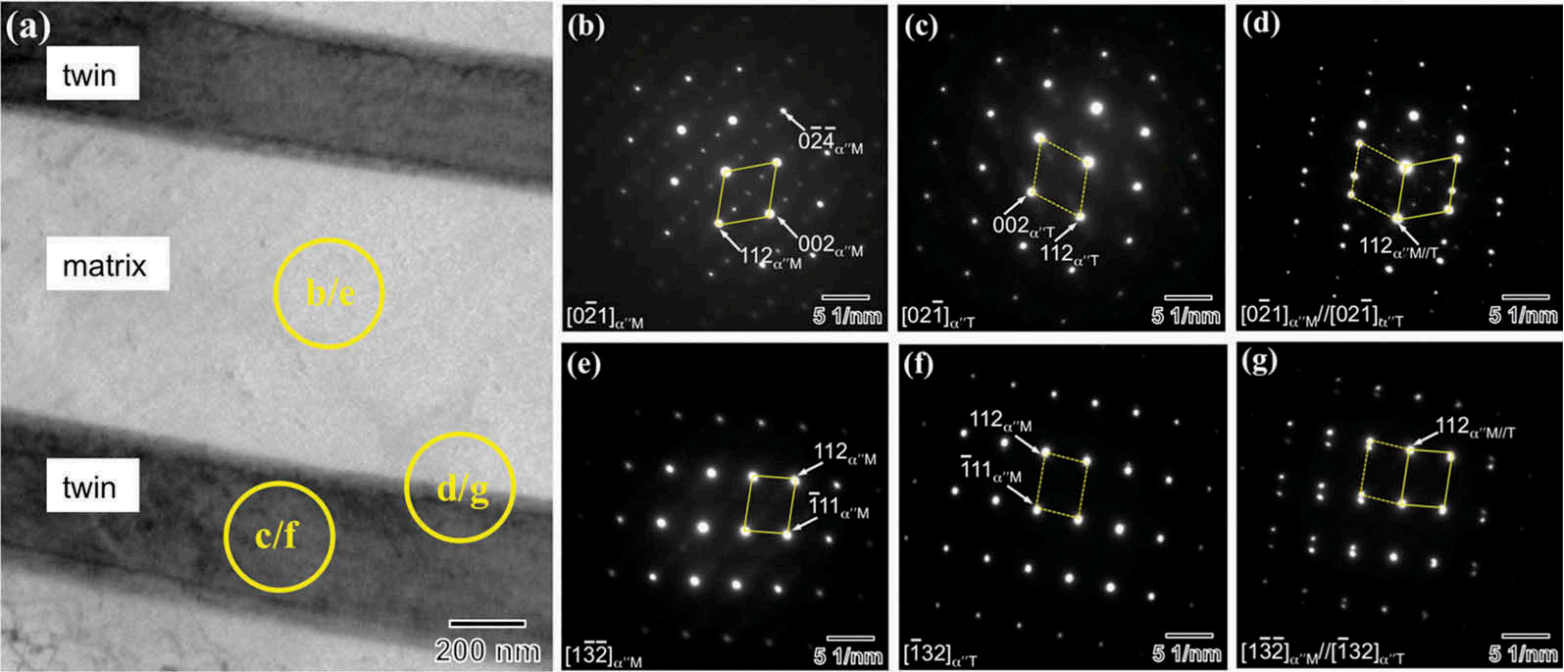
(a) TEM image of the lamella prepared by FIB from the location indicated in Figure 4(d). (b)-(g) SAED patterns taken from the circled regions indicated in (a).

**Figure 6.**
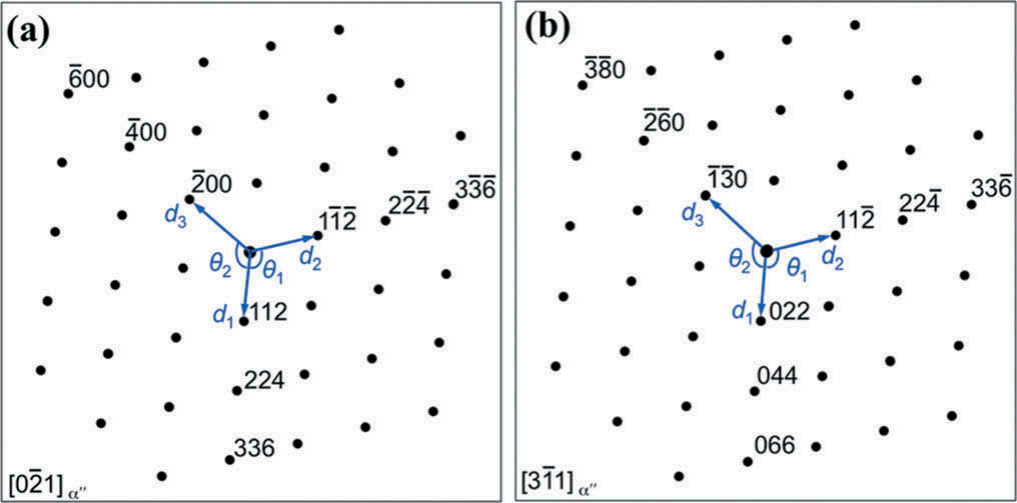
Calculated electron diffraction patterns along (a) [$0\overset{ˉ}{2}1$]_α”_ and (b) [$3\overset{ˉ}{1}1$]_α”_ zone axes with the lattice parameters of *a*_α”_ = 0.3002 nm, *b*_α”_ = 0.5033 nm, and *c*_α”_ = 0.4680 nm.

Deformation bands were further characterized by TEM on a FIB lift-out sample prepared in the cross-section perpendicular to the sample surface, Figure 4(b). Figure 5(a) shows a low magnified TEM image of variant V2. A twinning relationship between the deformation bands and crystal matrix can be obtained from the SAED patterns taken from the matrix (Figure 5(b,e)), twin (Figure 5(c,f)), and matrix/twin interface (Figure 5(d,g)) regions. However, the orthorhombic-αʺ martensite phase is a low symmetry crystal structure, which makes the unambiguous indexing of the SAED patterns a challenging task. In particular, the SAED patterns of Figure 5(b–d) can be indexed as {112}_αʺ_-type I twinning along the <$0\overset{ˉ}{2}1$>_αʺ_ zone axis and also as {011}_αʺ_-compound twinning along the <$3\overset{ˉ}{1}1$>_αʺ_ zone axis [37]. Similarly, the SAED patterns of Figure 5(e,g) are consistent with the diffraction patterns of {112}_αʺ_-type I twinning along the <$1\overset{ˉ}{3}\overset{ˉ}{2}$>_αʺ_ zone axis and also with those of {011}_αʺ_-compound twinning along the <$2\overset{ˉ}{1}1$>_αʺ_ zone axis. In order to unambiguously determine the active twinning system, we have performed a detailed analysis of the experimental SEAD pattern of Figure 5(b) as follows. The calculated electron diffraction patterns along the <$0\overset{ˉ}{2}1$>_αʺ_ and <$3\overset{ˉ}{1}1$>_αʺ_ zone axes for the current microscope conditions are shown in Figure 6(a,b), respectively. The diffraction patterns are characterized by a set of crystallographic parameters, namely interplanar spacings (*d*_2_ */d*_1_, *d*_3_ */d*_1_) of corresponding lattice planes and the angles (*θ*_1_, *θ*_2_) between these lattice planes, Table 1. We have evaluated the difference between the set of crystallographic parameters of the experimental diffraction pattern of Figure 5(b) and those of the calculated diffraction patterns along the <$0\overset{ˉ}{2}1$>_αʺ_ (Figure 6(a)) and <$3\overset{ˉ}{1}1$>_αʺ_ (Figure 6(b)) zone axes by the Pearson’s chi-squared statistic parameter χ^2^ [40]. The parameter IQ = 1-χ^2^ estimated the indexing quality of the diffraction pattern to a given crystallographic direction. We obtain IQ = 0.997 for the <$0\overset{ˉ}{2}1$>_αʺ_ crystallographic direction and IQ = 0.973 for the <$3\overset{ˉ}{1}1$>_αʺ_ crystallographic direction. These results indicate that the experimental pattern of Figure 5(b) can be indexed as the <$0\overset{ˉ}{2}1$>_αʺ_ zone axis (Figure 6(a)) and hence the deformation twinning system activated in the orthorhombic-αʺ martensite corresponds to {112}_αʺ_-type I deformation twinning. Interestingly, extra weak spots at 1/2 {002}_αʺ_ and 1/4 {$0\overset{ˉ}{2}\overset{ˉ}{4}$}_αʺ_ positions are visible in the SAED patterns of Figure 5(b–d) along the <$0\overset{ˉ}{2}1$>_αʺ_ zone axis, which can be ascribed to the occurrence of an internal structure in αʺ martensite. The formation of such internal structure is currently under investigation.

**Table 1.** The ratios of interplanar spacings of corresponding lattice planes (*d*_2_ */d*_1_ and *d*_3_ */d*_1_) and the angles (*θ*_1_ and *θ*_2_) between these lattice planes indicated in Figure 6. The values are measured from experimental SAED pattern of Figure 5(b), calculated diffraction patterns along [$0\overset{ˉ}{2}1$]_α_ʺ (Figure 6(a)) and [$3\overset{ˉ}{1}1$]_αʺ_ (Figure 6(b)) zone axes.

	$d_{2}/d_{1}$	$d_{3}/d_{1}$	$\theta_{1}$	$\theta_{2}$
Experimental Figure 5(b)	0.996	0.862	109.0°	125.1°
[$0\overset{ˉ}{2}1$]_α”_-axis Figure 6(a)	1.000	0.866	109.6°	125.2°
[$3\overset{ˉ}{1}1$]_α”_-axis Figure 6(b)	1.011	0.855	107.9°	126.5°

## 4. Discussion

The present study reveals that two different twinning modes are activated in orthorhombic-αʺ martensite structure, that is, {111}_αʺ_-type I transformation twinning and {112}_αʺ_-type I deformation twinning. To the authors’ knowledge, {112}_αʺ_-type I deformation twinning has never been reported as active twinning modes in αʺ martensite in β-Ti alloys. Crystallographic analysis of the observed twinning modes, and in particular the newly reported {112}_αʺ_-type I twinning, will be discussed in the following sections.

### 4.1. {111}_α”_-type I transformation twinning

From crystallographic standpoint, transformation twin structure occurs as a result of a lattice invariant shear (LIS) to accommodate the martensitic transformation strain [41,42]. Several transformation twinning modes have been reported in orthorhombic-αʺ martensite structure, namely, {111}_αʺ_-type I [19,22–24], <211>_αʺ_-type II [19,25], and {011}_αʺ_-compound twinning [19,26,27]. In particular, Inamura et al. [19] have proposed an approach to predict the transformation twinning system for LIS by the infinitesimal deformation theory [43]. In this approach, the principal strains of the lattice correspondence variant between β and αʺ are given as follows:

(1) $$X_{1}=\frac{a_{\alpha^{\prime \prime }}-a_{\beta }}{a_{\beta }}$$

(2) $$X_{2}=\frac{b_{\alpha^{\prime \prime }}-\sqrt{2}a_{\beta }}{\sqrt{2}a_{\beta }}$$

(3) $$X_{3}=\frac{c_{\alpha^{\prime \prime }}-\sqrt{2}a_{\beta }}{\sqrt{2}a_{\beta }}$$

where *a*_αʺ_, *b*_αʺ_, *c*_αʺ_, are the lattice parameters of αʺ martensite and *a*_β_ is the lattice parameter of β phase. This approach suggests that transformation twinning mode depends on the sign of *Χ*_3_, namely, {111}_αʺ_-type I twinning and <211>_αʺ_-type II twinning are activated when *Χ*_3_ > 0, and {011}_αʺ_-compound twinning is activated when *Χ*_3_ < 0. From the experimental standpoint, *Χ*_3_ > 0 condition predicts the activation of {111}_αʺ_-type I twinning since <211>_αʺ_-type II twinning has been rarely observed in previous reports [19,28,44]. In the present alloy, we obtain *Χ*_3_ = 0.011 with characteristic lattice parameters (*c*_αʺ_ = 0.4680 nm; *a*_β_ = 0.3272 nm [45]). This condition sets the activation of {111}_αʺ_-type I twinning, which agrees with the present results (Figure 3).

### 4.2. {112}_α”_-type I deformation twinning

Crystallographic analysis of deformation twinning is commonly evaluated by the Bilby-Crocker theory [36]. This approach has been widely used to analyze the crystallographic characteristics of twinning modes in different lattice structures such as fcc [46], bcc [47], hcp [48], and low symmetry structures [27,37]. In this approach, deformation twinning is described by four crystallographic elements, namely, *K*_1_, *K*_2_, *η*_1_, and *η*_2_, where *K*_1_ is the twin plane, *η*_1_ is the twin direction, *K*_2_ is the reciprocal or conjugate twin plane, and *η*_2_ is the reciprocal or conjugate twin direction. In the present case, *K*_1_ = {112} was experimentally confirmed, Figure 5. The other twin elements (*K*_2_, *η*_1_, and *η*_2_) of the {112}_αʺ_-type I twinning were calculated with the lattice parameters of αʺ martensite in the Ti-7.5Mo alloy. The determined crystallographic elements are *K*_1_ = {112}, *η*_1_ = <$\bar{6.463},\;\overset{ˉ}{1},\;3.732$>, *K*_2_ = {$\bar{2.586},\;\overset{ˉ}{1},4.379$}, and *η*_2_ = <312>. It is noted that the *K*_2_ and *η*_1_ elements have irrational Miller indices for {112}_αʺ_-type I twinning.

**Figure 7.**
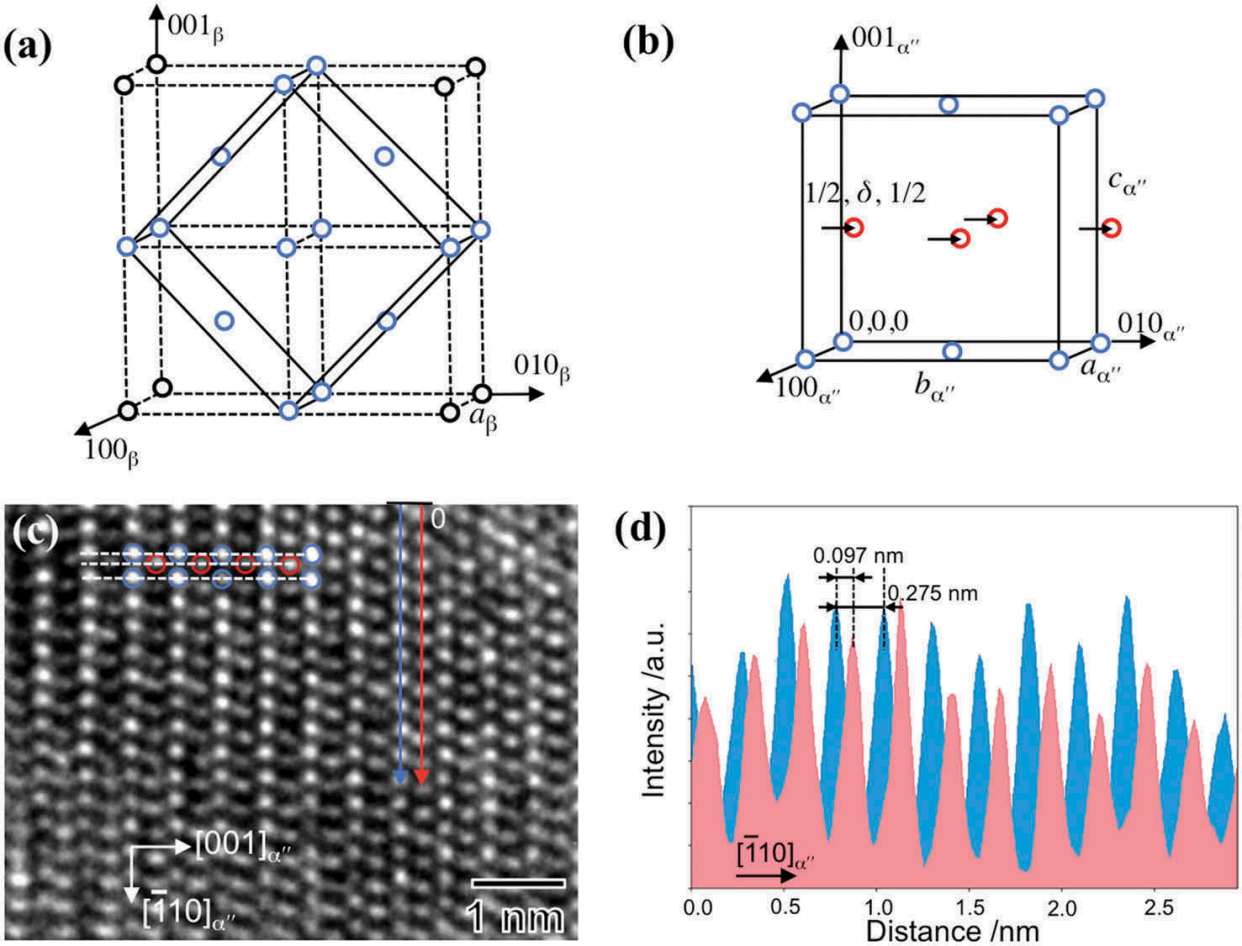
(a) The Au-Cd type lattice correspondence between β phase and αʺ phase [20]. The atoms involved in the formation of αʺ phase are shown in blue circles while the corner atoms are black circles. (b) Orthorhombic structure of αʺ martensite reported by Brown et al. [17]. The arrows and red circles indicate the shift of atoms on martensitic transformation. (c) HRTEM image of the as-quenched αʺ martensite along [110]_α“_ zone axis. (d) Intensity profile along the lines indicated in (c). The value of *δ* is evaluated by 1/2 − 0.097/0.275 ≈ 0.15.

The lattice correspondence between bcc-β and orthorhombic-αʺ martensite in β-Ti alloys is similar to that in Au-Cd alloys (Figure 7(a)) [19]. The atoms at face-centered positions in the orthorhombic lattice (red circles) are shifted to complete the β → αʺ martensitic transformation, Figure 7(b) [17,49]. The magnitude of this atomic shift is *δ* along the *b*-axis (black arrows in Figure 7(b)). Atoms are located at (0, 0, 0), (1/2, 1/2, 0), (0, 1/2 + *δ*, 1/2), and (1/2, *δ*, 1/2). The shifted atoms at positions of (1/2, *δ*, 1/2) result in the interpenetration of two orthorhombic lattices. From this finding, we consider the disordered orthorhombic-αʺ structure as a double lattice structure in the present study. It consists of motif units including two atoms arranged symmetrically about the lattice points of a single Bravais space lattice. According to previous reports [8,18], orthorhombic-αʺ martensite in β-Ti alloys can be described as a transitional phase between hcp-α*'* (*δ* = 1/6) and bcc-β (*δ* = 0). The magnitude of *δ* in αʺ structure is restricted to 0 < *δ* < 1/6. The atomic lattice structure of the as-quenched αʺ martensite is shown in the high-resolution TEM (HRTEM) image of Figure 7(c). From the intensity profile (Figure 7(d)), we obtained *δ* = 0.15. This result agrees with the value of *δ* (~0.157) for Ti-7.5Mo alloy determined by Li et al. [18] using first-principles calculation.

**Figure 8.**
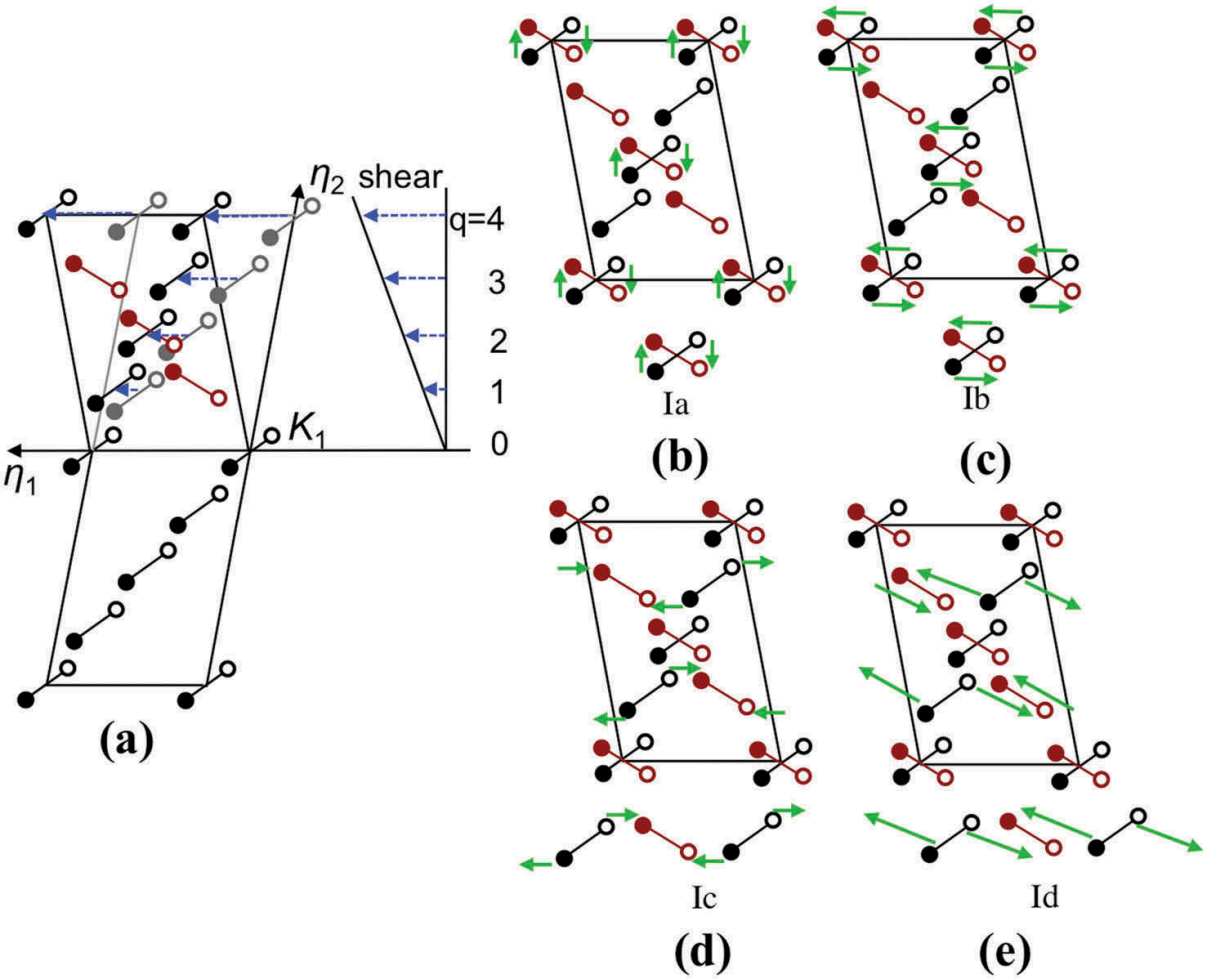
Schematically illustration of twinning modes with *q* = 4 in double lattice structures [37]. Shear of the lattice points (a). Shuffle mechanisms corresponding to the rearrangement of the motif units to form type I twin (b) and (c), and the disruption of the motif units to form type I twin (d) and (e). The atoms represented by closed and open circles lie above and below the paper, respectively.

**Figure 9.**
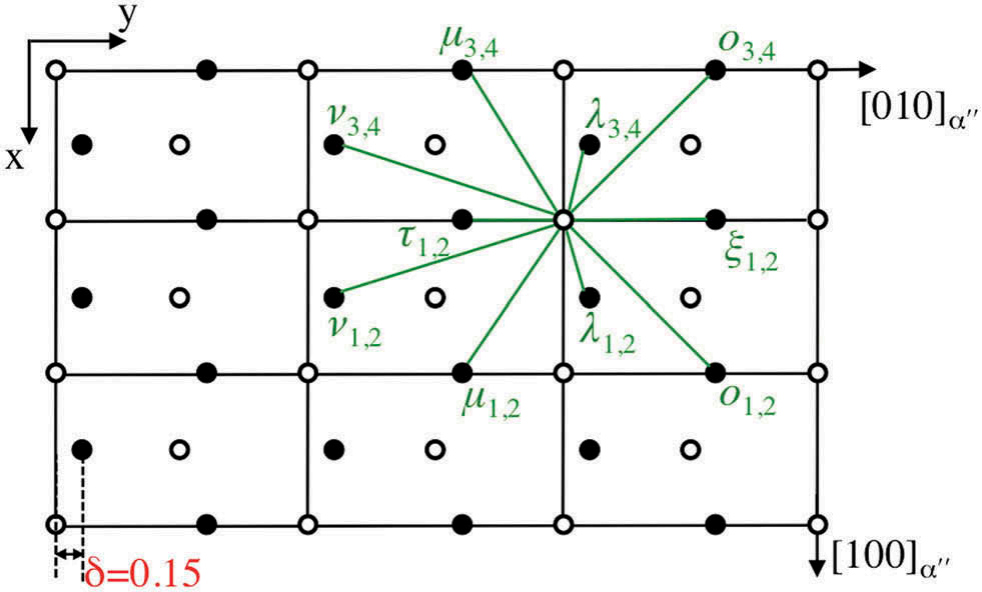
(001)_α“_ Projection of the possible motif units. Open circles represent atoms in the projection plane, and close circles are atoms above or below the projection.

**Figure 10.**
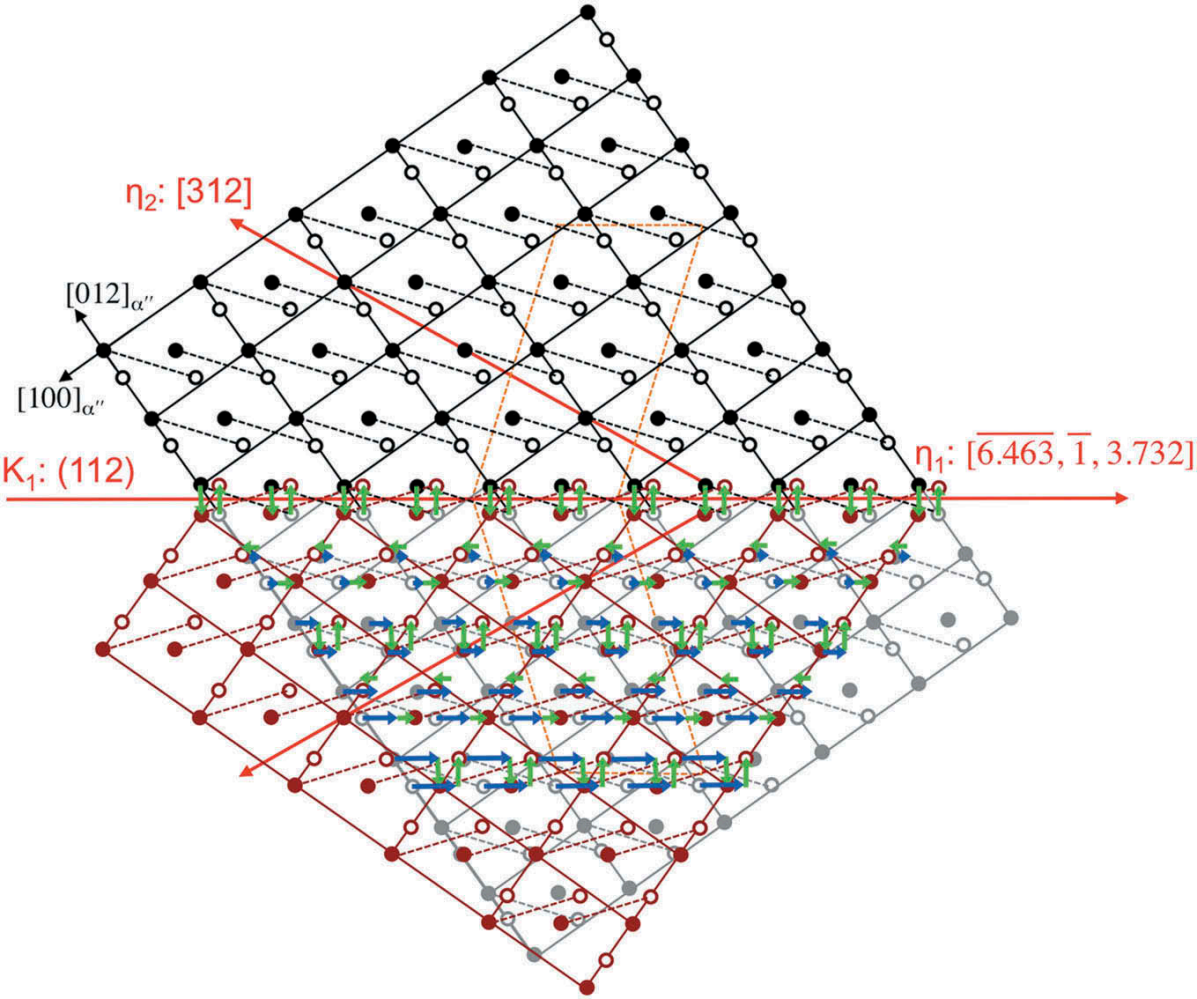
($02\overset{ˉ}{1}$)_αʺ_ projection of the most possible atomic movement of {112}_αʺ_-type I twinning in the Ti-7.5Mo alloy. Open circles represent atoms in the projection plane, and close circles are atoms above or below the projection. The dashed lines indicate the motif units. The blue arrows indicate homogeneous atomic shears comprising the units. The green arrows indicate atomic shuffles with the mechanisms Ia and Ic for different planes.

According to the Bilby-Crocker theory, the atomic movements accompanying the deformation twinning involve shear and shuffle components. The motif pairs of atoms in double lattice structures are assumed to shear homogeneously as rigid bodies. Taking into account that the shear magnitude *s* is given by *s* = 2 cot (2*ϕ)*, where (2*ϕ)* is the angle between *K*_1_ and *K*_2_ planes, we obtained *s* = 0.199 of the present {112}_αʺ_-type I twinning. Moreover, additional atomic shuffles must accompany the shears to reach to the twin positions. The complexity of shuffle mechanisms is directly related to the number of lattice planes *q* parallel to *K*_1_ that intersected by *η*_2_ for type I twinning. In the present twinning mode, we obtained *q* = 4 [37]. The shuffle mechanisms corresponding to *q* = 4 for type I twinning in the double lattice structure are illustrated in Figure 8. The homogeneous shear components are indicated with blue arrows in Figure 8(a), which increase from zero on the composition plane (designed plane 0). The rearrangement of motif units at the even planes, including the composition plane, must take place by means of shuffle mechanisms Ia (Figure 8(b)) or Ib (Figure 8(c)). Meanwhile, disruption of motif units on the odd lattice planes is also required by means of shuffle mechanism Ic (Figure 8(d)) or Id (Figure 8(e)). According to this model, the most favored atomic shuffle mechanism is suggested corresponding to those with the smallest shuffle magnitude (*Δ*) [36]. From crystallographic standpoint, there are six distinct pairs of atoms comprising the motif unit in the disordered orthorhombic-αʺ structure, namely *κ, λ, μ, ν, ο*, and *ξ* [37]. The projection of motif units on the (001) plane is shown in Figure 9. The units of *κ* and *ξ* have two equivalent variants and the units of *λ, μ, ο*, and *ν* have four each. Accordingly, there are a total of 20 possible units, Table 2. The smallest possible motif units lying approximately parallel to the *K*_1_ plane and resulting in small shuffle magnitudes has been adopted to choose the motif unit [37,47]. Consequently, it is set as *λ*_2_. The calculation method for the shuffle magnitudes is described in Appendix (Table A1). According to the results, the motif unit of {112}_αʺ_-type I twinning is set as *λ*_2_ with the shuffle magnitude *Δ*_Ia_ = 0.61 Å for Ia, and *Δ*_Ic_ = 1.25 Å for Ic. As a result, the most possible mechanism of atomic movements including shears and shuffles of {112}_αʺ_-type I twinning is illustrated in Figure 10. It is noted that the twin plane is set in the middle of the two planes in order to have smaller interplanar spacing. Homogenous shears are indicated with blue arrows, while the shuffle mechanisms including Ia on even planes and Ic on the odd planes are indicated with green arrows. The above crystallographic analysis enables an adequate understanding of the newly reported {112}_αʺ_-type I twinning in orthorhombic-αʺ martensite.

**Table 2.** Possible motif units: [*xyz*].

Unit	*x*	*y*	*z*
*λ*_1_	0.50	0.15	0.50
*λ*_2_	0.50	0.15	−0.50
*λ*_3_	−0.50	0.15	0.50
*λ*_4_	−0.50	0.15	−0.50
*τ*_1_	0.00	−0.35	0.50
*τ*_2_	0.00	−0.35	−0.50
*ξ*_1_	0.00	0.65	0.50
*ξ*_2_	0.00	0.65	−0.50
*μ*_1_	1.00	−0.35	0.50
*μ*_2_	1.00	−0.35	−0.50
*μ*_3_	−1.00	−0.35	0.50
*μ*_4_	−1.00	−0.35	−0.50
*ο*_1_	1.00	0.65	0.50
*ο*_2_	1.00	0.65	−0.50
*ο*_3_	−1.00	0.65	0.50
*ο*_4_	−1.00	0.65	−0.50
*ν*_1_	0.50	−0.85	0.50
*ν*_2_	0.50	−0.85	−0.50
*ν*_3_	−0.50	−0.85	0.50
*ν*_4_	−0.50	−0.85	−0.50

The twinning mode observed in α“ martensite in the present study, i.e., {112}_αʺ_-type I twinning, has not been reported in β-Ti alloys so far. However, this twinning mode has been frequently reported in α-uranium [50,51], which also contains orthorhombic structure. Owing to the low symmetry of orthorhombic structure, plastic deformation is accommodated by the activation of multiple twinning modes [37]. In particular, five of the 82 possible twinning modes predicted by the Bilby-Crocker theory in α-uranium have been experimentally observed [37,50,52,53]. In β-Ti alloys, several studies have analyzed the twinning behavior of αʺ martensite [26,36,45]. These works have reported the activation of different twinning modes with *s* ~ 0.2–0.3, which is close to the shear magnitude of the {112}_αʺ_-type I twinning mode (*s* = 0.199). Our experimental results (Figure 4–5) indicate that {112}_αʺ_-type I deformation twinning is activated in the present αʺ martensite of Ti-7.5Mo alloy. However, due to the low crystal symmetry of αʺ martensite, other twinning modes can be activated as well.

## 5. Conclusions

We have investigated the as-quenched and deformation microstructures of orthorhombic-αʺ martensite in a Ti-7.5Mo (wt.%) alloy by the combined use of scanning electron microscopy (SEM) and transmission electron microscopy (TEM). The main conclusions are as follows:

- The as-quenched αʺ martensite plates contained an internal twin structure of {111}_αʺ_-type I transformation twins, which is ascribed to β → αʺ martensitic transformation strain accommodation.

- Activation of {112}_αʺ_-type I deformation twinning was identified for the first time in orthorhombic-αʺ martensite in β-Ti alloys upon tensile straining.
- {112}_αʺ_-type I deformation twinning has been analyzed by the crystallographic theory of deformation twinning by Bilby and Crocker. The most possible mechanism of atomic displacements involving shears and shuffles has been proposed.

## Appendix Appendix

Table A1 shows the equations to calculate the shuffle magnitudes [37]. (*hkl*) are the Miller indices of the twin plane *K*_1_ for type I twinning and (*abc*) are those of the twin direction *η*_1_ for type II twinning. [*xyz*] represent the vectors of the motif units in Table 1. In order the specify the shuffle magnitudes of Ic and Id modes, in which *η*_1_ is irrational, a lattice vector [*efg*] lying in *K*_1_ is chosen to define the relative positions of the parent motif units. In this condition, *he* + *kf* + *lg *= 0 must be satisfied. In order to involve the smallest shuffle magnitudes, we set the vector [*efg*] to be $\left[10\bar{\frac{1}{2}}\right]$ for {112}_αʺ_-type I twinning.

**Table A1.** Equations for shuffle magnitudes in orthorhombic double lattice structure [37].

Shuffle	Magnitude
Ia	$(hx+ky+lz)(h2a_{\alpha^{\prime \prime }}^{-2}+k2b_{\alpha^{\prime \prime }}^{-2}+l2c_{\alpha^{\prime \prime }}^{-2}{)}^{-1/2}=A$
Ib	$[(x2a_{\alpha^{\prime \prime }}^{2}+y2b_{\alpha^{\prime \prime }}^{2}+z2c_{\alpha^{\prime \prime }}^{2})-A^{2}{]}^{1/2}$
Ic	${\left\{[(e-2x)2a_{\alpha^{\prime \prime }}^{2}+(f-2y)2b_{\alpha^{\prime \prime }}^{2}+(g-2z)2c_{\alpha^{\prime \prime }}^{2}]/4-A^{2}\right\}}^{1/2}$
Id	$[(e2a_{\alpha^{\prime \prime }}^{2}+f2b_{\alpha^{\prime \prime }}^{2}+g2c_{\alpha^{\prime \prime }}^{2})-A^{2}{]}^{1/2}$
